# Safety of non-standard regimen of systemic steroid therapy in patients with Graves’ orbitopathy: a single-centre experience

**DOI:** 10.1007/s43440-023-00567-0

**Published:** 2024-01-26

**Authors:** Nadia Sawicka-Gutaj, Dawid Gruszczyński, Natalia Zawalna, Kacper Nijakowski, Agnieszka Skiba, Mateusz Pochylski, Jerzy Sowiński, Marek Ruchała

**Affiliations:** 1 Department of Endocrinology, Metabolic Disorders and Internal Medicine, Poznań University of Medical Sciences, 49 Przybyszewskiego Street, 60-355 Poznań, Poland; 2 Department of Conservative Dentistry and Endodontics, Poznań University of Medical Sciences, Poznań, Poland

**Keywords:** Glucocorticoids, Graves’ orbitopathy, Hyperglycemia, Intravenous methylprednisolone, Side effects

## Abstract

**Background:**

Graves’ orbitopathy (GO) is an autoimmune disorder of the orbit and retro-ocular tissues and the primary extrathyroidal manifestation of Graves’ disease. In moderate-to-severe and active GO *iv* glucocorticoids (GCs) are recommended as first-line treatment. The aim was to assess the safety profile of methylprednisolone administered intravenously for three consecutive days at 1 g in patients with active, moderate-to-severe or sight-threatening Graves’ orbitopathy.

**Methods:**

We retrospectively evaluated 161 medical records of patients with GO treated with high-dose systemic GCs in the Department of Endocrinology, Metabolic Disorders, and Internal Medicine in Poznań between 2014 and 2021. Clinical data included age, gender, laboratory results, activity and severity of GO, smoking status, disease duration, and presented side effects.

**Results:**

The presence of mild side effects was observed during 114 (71%) hospitalizations. The most common complications were hyperglycemia (*n* = 95) and elevated aminotransferases (*n* = 31). Increased levels of aminotransferases were more likely observed in smokers and GO duration above 12 months. Based on the multivariate logistic regression, higher TRAb and CAS values were significantly associated with lower odds of hyperglycemia. In turn, the increased odds of elevated aminotransferases were significantly correlated with higher initial ALT levels, female gender, and GO duration above 12 months. In addition, the multidimensional correspondence analysis (MPA) showed that GO patients who declared smoking and had not l-ornithine l-aspartate applied demonstrated a higher probability of elevated aminotransferases.

**Conclusions:**

Active GO treatment with high-dose systemic GCs is not associated with serious side effects. Hyperglycemia is the most common steroid-induced complication.

## Introduction

Graves’ orbitopathy (GO), also called Graves’ ophthalmopathy, thyroid-associated ophthalmopathy (TAO), or thyroid eye disease (TED), is an autoimmune disorder of the orbit and retrobulbar tissues, representing the major extrathyroidal manifestation of Graves’ disease (GD) [1–3]. Although GO is most frequently associated with hyperthyroidism, it may rarely occur in euthyroid or hypothyroid patients [3, 4]. App. 6% of GD patients demonstrate moderate-to-severe active form. In addition, the sight-threatening GO with dysthyroid optic neuropathy (DON) is observed in fewer than 1% of patients [2]. Full-blown disease is associated with disfiguring features, inflammatory signs and symptoms, visual disturbances, and may lead to permanent visual loss. These characteristic manifestations significantly reduce patients’ health-related quality of life [5–7].

Both endogenous and exogenous risk factors may affect the course and severity of GO. Among the non-modifiable factors are gender, age, race, and genetic susceptibility [5, 8, 9]. Although there is a predilection for the female gender, males are more prone to a severe course, especially at the age above 50 years. The environmental factors include thyroid dysfunction, radioactive iodine (RAI) treatment, oxidative stress, and hypercholesterolemia; however, the main modifiable factor is smoking exposure (active and passive) [5, 8, 9]. Tobacco smokers experience more exacerbated ocular symptoms and poorer response to standard immunosuppressive therapy [10, 11]. Elevated thyrotropin-receptor antibodies (TRAbs) titers predispose to similar clinical outcomes [11].

Management of patients with GO requires a multidisciplinary approach based on clinical activity, severity, and disease duration [12]. Also, general measures for all GO patients include eliminating modifiable risk factors. The “wait and watch” strategy and local treatments (artificial tears, gels or ointments and dark glasses) are usually sufficient in mild and active GO. Due to its anti-inflammatory and antioxidant properties, selenium supplementation for 6 months may benefit patients in selenium-deficient areas. In turn, intravenous glucocorticoid pulse therapy (*iv* GCs) in monotherapy or combined with mycophenolate sodium is considered the first‐line treatment for moderate-to-severe and active GO. The EUGOGO protocol recommends a total cumulative dose of 4.5 g methylprednisolone, given in 12 weekly infusions (0.5 g/week for six weeks, followed by 0.25 g/week for six weeks) [13, 14]. Although effective, this treatment may be accompanied by various adverse events (AEs), such as flushing, hypertension, hyperglycemia, arrhythmias, liver dysfunction, psychosis and infection [10, 15]. However, most reported AEs are associated with single and cumulative doses of methylprednisolone higher than recommended. Moreover, oral therapy is associated with higher steroid-related AEs, including weight gain, hypertension and cushingoid features [16–18]. EUGOGO protocol requires twelve hospital admissions, significantly deteriorating patients’ socioeconomic life in our region. Therefore, in 2010 the non-standard protocol was developed, and its safety profile and effectiveness have been investigated in a prospective study (PhD dissertation of Agnieszka Skiba under the supervision of Prof. Jerzy Sowiński).

Our study aimed to assess the safety profile of methylprednisolone administered intravenously for three consecutive days at a dose of 1 g in patients with active, moderate-to-severe or sight-threatening Graves’ orbitopathy.

## Materials and methods

### Patients

**Fig. 1:**
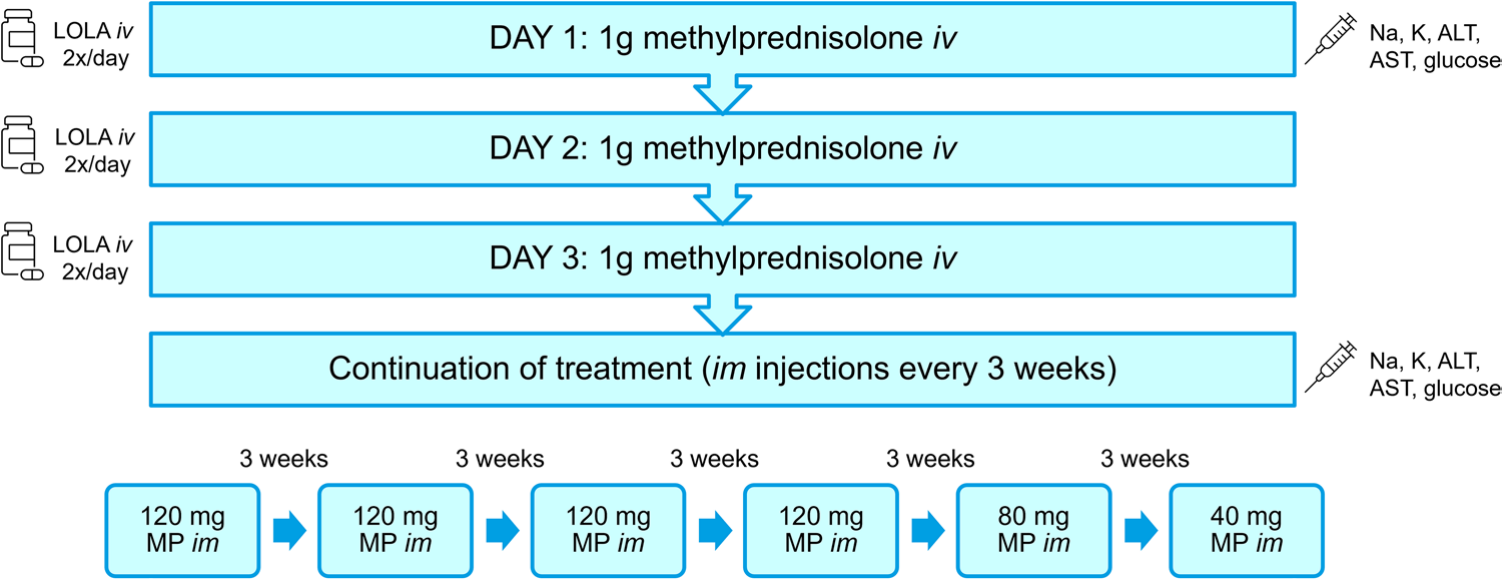
Non-standard regimen of systematic steroid therapy of Graves’ orbitopathy in Department of Endocrinology, Metabolic Disorders and Internal Medicine, Poznań University of Medical Sciences, Poznań, Poland. *ALT* alanine aminotransferase, *AST* aspartate aminotransferase, *im* intramuscular, *iv* intravenous, *LOLA* l-ornithine l-aspartate, *MP* methylprednisolone

We conducted a retrospective single-centre study evaluating 161 medical records of patients with active, moderate-to-severe and sight-threatening GO treated with high-dose systemic glucocorticoids in the Department of Endocrinology, Metabolic Disorders and Internal Medicine at Poznań University of Medical Sciences in 2014–2021. The treatment protocol consisted of *iv* methylprednisolone administered for three consecutive days at a dose of 1 g, followed by 600 mg *im* methylprednisolone in divided doses (cumulative dose, 3.6 g) — Fig. 1. Most patients also received l-ornithine l-aspartate (LOLA) and proton pump inhibitor (PPI) during hospitalization.

During qualification for this treatment, each patient underwent a physical examination, followed by an ophthalmological consultation and magnetic resonance imaging of orbits. The clinical activity score (CAS) was used to assess GO severity. Moreover, laboratory tests were performed before and after the *iv* GCs administration.

Data were obtained from the electronic medical records of the hospital. Clinical data included age, gender, activity and severity of GO, smoking status, duration of the disease, and presented side effects. Also, we collected laboratory results, including Na, K, alanine aminotransferase (ALT), aspartate aminotransferase (AST), thyroid-stimulating hormone (TSH), free triiodothyronine (fT3), free thyroxine (fT4), thyroid-stimulating hormone receptor antibodies (TRAb), thyroid peroxidase antibodies (TPOAb), thyroglobulin antibodies (TgAb), glucose, C-reactive protein (CRP), anti-HCV, HBsAg.

Hyperglycemia was a fasting plasma glucose level of ≥ 100 mg/dL and ≥ 140 mg/dL two hours after meal. We analyzed steroid-induced changes in aminotransferase levels; however, clinically significant aminotransferase increase was defined as ALT or AST elevation by > 3 × the upper limit of normal (ULN).

### Ethics statement

We adhered to the ethical standards of the Declaration of Helsinki when we collected, analyzed and reported the data [19]. Due to the retrospective design, the bioethics committee approval is not required.

### Statistical analysis

Due to the non-compliance of the continuous variables to the normal distribution (assessed by the Shapiro–Wilk test), the comparisons were made using the Mann–Whitney test or the Wilcoxon test. The Pearson’s Chi-squared test compared distributions of qualitative variables. Univariate and multivariate logistic regression modelling was conducted, describing the odds for the major complications. Also, MPA was used to assess the relationship between major complications and qualitative variables. The significance level was set at *α* = 0.05 for all analyses. Statistical analysis was performed with Statistica 13.3 (Statsoft, Cracow, Poland).

## Results

### Characteristics of patients

The analyzed records included patients with moderate-to-severe (*n* = 110) and sight-threatening (*n* = 51) Graves’ orbitopathy. Table 1 presents detailed characteristics of recruited patients.

**Table 1:** Detailed characteristics of records in patients with Graves’ orbitopathy recruited to non-standard regimen of systematic steroid therapy in Department of Endocrinology, Metabolic Disorders and Internal Medicine, Poznań University of Medical Sciences, Poznań, Poland

	*n* = 161
Sex (female), *n* (%)	114 (70.8)
Age, years	56 (46–63)
BMI, kg/m^2^	26.7 (23.1–29.1)
Smoking, *n* (%)	59 (36.6)
Concomitant diseases, *n* (%)	116 (72.0)
Disease duration, months	12 (6–23)
Previous *iv* glucocorticoids, *n* (%)	40 (24.8)
LOLA supplementation, *n* (%)	115 (71.4)
TSH, µU/mL	0.97 (0.31–2.33)
fT3, pmol/L	4.38 (3.93–4.99)
fT4, pmol/L	18.60 (15.30–20.38)
TRAb, IU/L	7.09 (2.26–19.16)
TPOAb, IU/mL	31 (12–165)
TgAb, IU/mL	16 (11–126)
CAS:	4 (3–5)
Spontaneous retrobulbar pain, *n* (%)	68 (42.2)
Pain on attempted upward or downward gaze, *n* (%)	70 (43.5)
Redness of eyelids, *n* (%)	62 (38.5)
Redness of conjunctiva, *n* (%)	110 (68.3)
Swelling of caruncle or plica, *n* (%)	119 (73.9)
Swelling of eyelids, *n* (%)	72 (44.7)
Swelling of conjunctiva (chemosis), *n* (%)	62 (38.5)

Continuous data presented as median and quartile ranges

*BMI* body mass index, *CAS* clinical activity score, *fT3* free triiodothyronine, *fT4* free thyroxine, *iv* intravenous, *LOLA* l-ornithine l-aspartate, *TgAb* thyroglobulin antibodies, *TPOAb* thyroid peroxidase antibodies, *TRAb* thyroid-stimulating hormone receptor antibodies, *TSH* thyroid-stimulating hormone

Among the concomitant diseases, the most common were cardiovascular diseases (*n* = 61), hyperlipidemia (*n* = 42) and diabetes mellitus (*n* = 14). Interestingly, diabetes did not affect the presence of steroid-induced hyperglycemia (*χ*^2^ = 1.884, df = 1, *p* = 0.170, Pearson’s Chi-squared test) and elevated TRAb levels (*χ*^2^ = 0.370, df = 1, *p* = 0.543, Pearson’s Chi-squared test).

### Comparison of biochemical parameters before and after intravenous steroid therapy

For the whole group, as a result of steroid therapy, significantly increased levels of Na, glucose and ALT, and decreased levels of K and AST were observed (Table 2). Only two patients demonstrated levels of aminotransferases higher than three times the ULN.

**Table 2:** Comparison of biochemical parameters before and after intravenous steroid therapy in patients with Graves’ orbitopathy—Wilcoxon test (*significant difference)

	Before	After	*T*	*p*	*n*
Na, mmol/L	142	143	1559	< 0.001*	112
K, mmol/L	4.45	4.28	2164	< 0.001*	133
Glucose, mg/dL	96	119	867	< 0.001*	130
ALT, U/L	19	23	1927	< 0.001*	116
AST, U/L	19	17	2598	0.010*	119

*ALT* alanine aminotransferase, *AST* aspartate aminotransferase

Considering LOLA supplementation, pre-treatment AST and ALT levels were not different. However, in the LOLA group, post-treatment AST was significantly lowered (17 vs 21; U = 812, *p* = 0.033, *n*_1_ = 99, *n*_2_ = 23, Mann–Whitney test), and ALT was lowered with borderline significance (22.5 vs 28.5; U = 842, *p* = 0.086, *n*_1_ = 100, *n*_2_ = 22, Mann–Whitney test) compared to the non-LOLA group.

After the steroid therapy, ALT levels significantly increased in both groups but less in the LOLA group (4.5 vs 8.5; respectively, *T* = 1280.5, *p* < 0.001, *n* = 92, *T* = 44.5, *p* = 0.008, *n* = 22, Wilcoxon test). In contrast, a significant decrease in AST was found in the LOLA group (*T* = 1429.5, *p* = 0.004, *n* = 93, Wilcoxon test), without statistically significant changes in the non-LOLA group (*T* = 126, *p* = 0.715, *n* = 23, Wilcoxon test).

### Analyses of steroid therapy-induced side effects

Side effects due to steroid therapy were observed during 116 hospitalizations. The most common were hyperglycemia (*n* = 95), aminotransferase increase (*n* = 31), headache (*n* = 11), facial erythema (*n* = 11), abdominal pain (*n* = 5), blood pressure increase (*n* = 3), delirium (*n* = 1), resting tremor (*n* = 2), insomnia (*n* = 3), peripheral oedema (*n* = 2).

#### Distribution comparisons

A higher percentage of hyperglycemia occurred in non-smokers and with normal TRAb levels (at borderline statistical significance). A higher rate of elevated aminotransferases was found in smokers and patients with more than 12 months of disease duration. Also, patients treated for the first time with intravenous steroid therapy and supplemented LOLA appeared to have less often elevated aminotransferase levels (with borderline significance). Detailed results of comparisons are shown in Table 3.

**Table 3:** Distribution comparisons of main steroid therapy-induced side effects (hyperglycemia and aminotransferase increase) in patients with Graves’ orbitopathy depending on demographic and clinical factors—Pearson’s Chi-squared test, df = 1

	Hyperglycemia		Aminotransferase increase	
	Yes	No	Yes	No
Sex				
Female	61	34	23	65
Male	25	15	8	29
*p*, *χ*^2^	0.850, 0.036		0.594, 0.285	
BMI				
< 30 kg/m^2^	65	34	23	71
≥ 30 kg/m^2^	18	13	8	19
*p*, *χ*^2^	0.443, 0.589		0.588, 0.293	
Smoking				
Yes	56	27	17	31
No	30	21	14	62
*p*, *χ*^2^	0.311, 1.027		0.033, 4.532	
Disease duration				
≤ 12 months	37	23	8	45
> 12 months	47	23	22	45
*p*, *χ*^2^	0.515, 0.424		0.026, 4.968	
Previous *iv* glucocorticoids				
Yes	24	12	13	22
No	61	37	18	71
*p*, *χ*^2^	0.638, 0.222		0.050, 3.835	
LOLA supplementation				
Yes	19	12	22	78
No	64	37	9	13
*p*, *χ*^2^	0.834, 0.044		0.065, 3.402	
TRAb				
< 2 IU/L	23	7	6	24
≥ 2 IU/L	60	41	21	70
*p*, *χ*^2^	0.085, 2.968		0.726, 0.123	

*BMI* body mass index, *iv* intravenous, *LOLA* l-ornithine l-aspartate, *TRAb* thyroid-stimulating hormone receptor antibodies

#### Logistic regression

Logistic regression modelling was performed. Table 4 presents the predictors significant and borderline significant in the univariate analysis for hyperglycemia and elevated aminotransferases, respectively. Lower odds of hyperglycemia were significantly associated with higher CAS values and TRAb levels. Cigarette smoking and disease duration > 1 year indicated significantly more than twofold higher odds of elevated aminotransferases after steroid therapy.

**Table 4:** Parameters of significant and borderline significant predictors in the univariate logistic regression model describing odds for steroid-induced hyperglycemia and steroid-induced aminotransferase increase in patients with Graves’ orbitopathy

	*β*	SE	Walda Stat.	*p*	OR	−95% CI	95% CI
Steroid-induced hyperglycemia							
TRAb, IU/L	− 0.048	0.014	12.109	< 0.001*	0.953	0.928	0.979
CAS	− 0.327	0.116	7.883	0.005*	0.721	0.574	0.906
Steroid-induced aminotransferase increase							
ALT-1, U/L	0.079	0.022	13.295	< 0.001*	1.082	1.037	1.129
AST-1, U/L	0.091	0.039	5.355	0.021*	1.095	1.014	1.182
GO duration > 1y	1.012	0.464	4.762	0.029*	2.75	1.108	6.822
Smoking	0.887	0.423	4.407	0.036*	2.429	1.061	5.561
Previous *iv* glucocorticoids	0.846	0.438	3.729	0.054*	2.331	0.987	5.502
LOLA supplementation	− 0.898	0.496	3.274	0.070*	0.407	0.154	1.078

*ALT-1* alanine aminotransferase before steroid therapy, *AST-1* aspartate aminotransferase before steroid therapy, *CAS* clinical activity score, *CI* confidence interval, *GO* Graves’ orbitopathy, *iv* intravenous, *LOLA* l-ornithine l-aspartate, *OR* odds ratio, *SE* standard error, *TRAb* thyroid-stimulating hormone receptor antibodies

*Significant predictor in logistic regression model

Also, the multivariate regression models were constructed using the stepwise forward technique (Table 5). Again, higher levels of CAS and TRAb protected steroid-induced hyperglycemia (in V-fold cross-validation: training AUC = 0.740 and validation AUC = 0.716). In turn, the GO duration over a year increased the odds of elevated aminotransferases more than 3.5 times, and the male sex decreased by nearly 90% (in V-fold cross-validation: training AUC = 0.842 and validation AUC = 0.799).

**Table 5:** Parameters of predictors incorporated into the multivariate logistic regression model (using the stepwise forward technique) describing odds for steroid-induced hyperglycemia and steroid-induced aminotransferase increase in patients with Graves’ orbitopathy

	*β*	SE	Walda Stat.	*p*	OR	− 95% CI	95% CI
Steroid-induced hyperglycemia							
Intercept	3.021	0.692	19.08	< 0.001*			
TRAb, IU/L	− 0.05	0.015	10.97	0.001*	0.951	0.923	0.98
CAS	− 0.31	0.128	5.856	0.016*	0.734	0.571	0.943
Steroid-induced aminotransferase increase							
Intercept	− 4.655	1.026	20.571	< 0.001*			
ALT-1, U/L	0.149	0.038	15.248	< 0.001*	1.161	1.077	1.251
Male sex	− 2.158	0.823	6.87	0.009*	0.116	0.023	0.58
GO duration > 1y	1.283	0.637	4.057	0.044*	3.608	1.035	12.578

*ALT-1* alanine aminotransferase before steroid therapy, *CAS* clinical activity score, *CI* confidence interval, *GO* Graves’ orbitopathy, *OR* odds ratio, *SE* standard error, *TRAb* thyroid-stimulating hormone receptor antibodies

*Significant predictor in logistic regression model

#### Multidimensional correspondence analysis

**Fig. 2:**
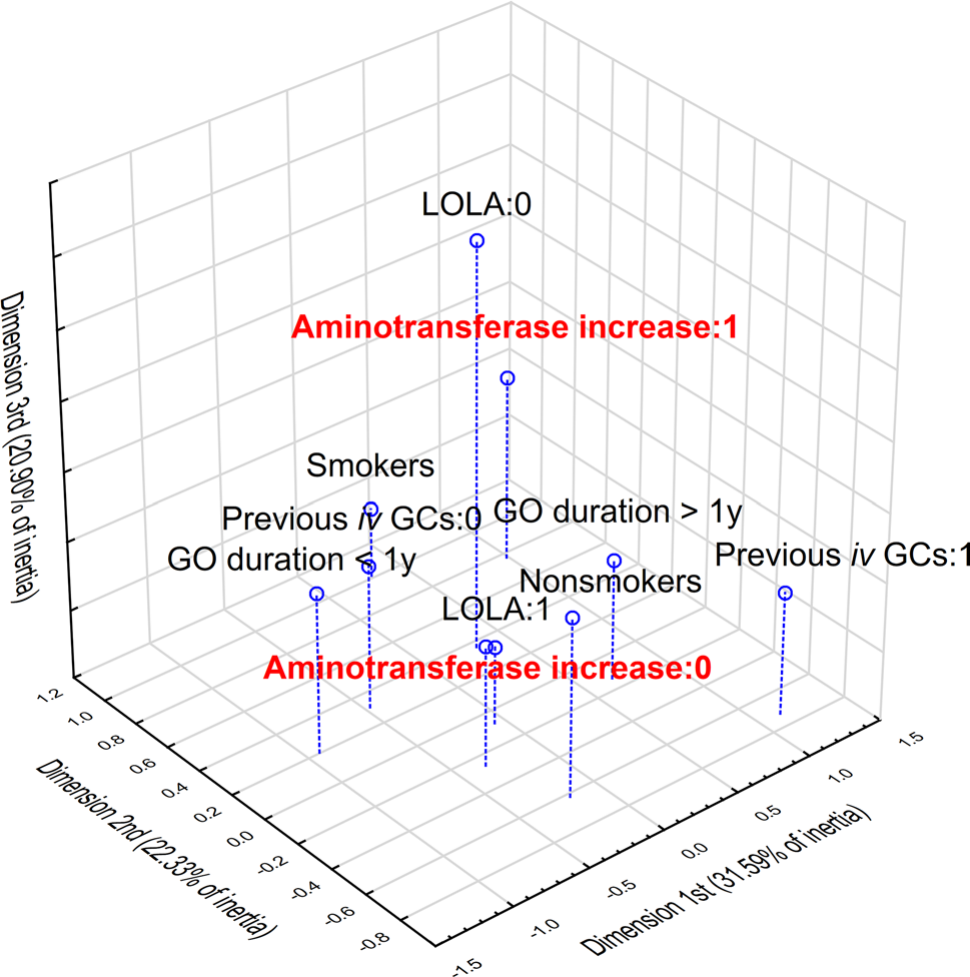
Multidimensional correspondence analysis for steroid-induced aminotransferase increase in patients with Graves’ orbitopathy—3-dimensional plot. *GCs* glucocorticoids, *GO* Graves’ orbitopathy, *iv* intravenous, *LOLA* l-ornithine l-aspartate

**Fig. 3:**
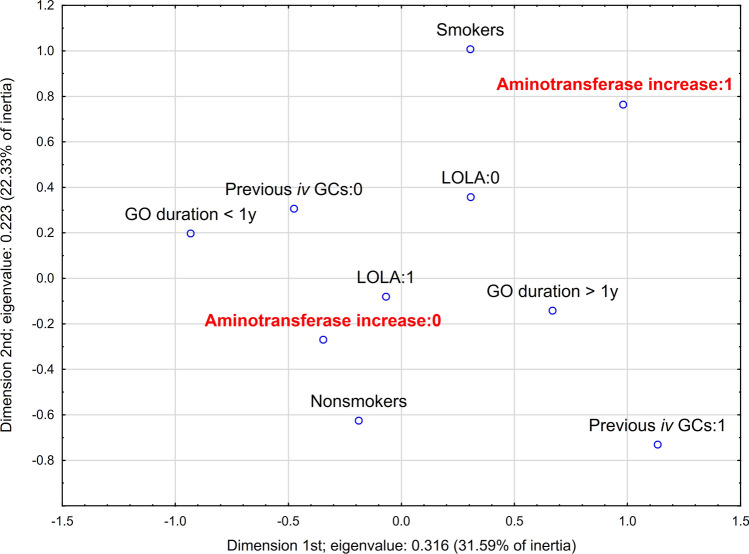
Multidimensional correspondence analysis for steroid-induced aminotransferase increase in patients with Graves’ orbitopathy—2-dimensional plot with the highest inertias. *GCs* glucocorticoids, *GO* Graves’ orbitopathy, *iv* intravenous, *LOLA* l-ornithine l-aspartate

**Fig. 4:**
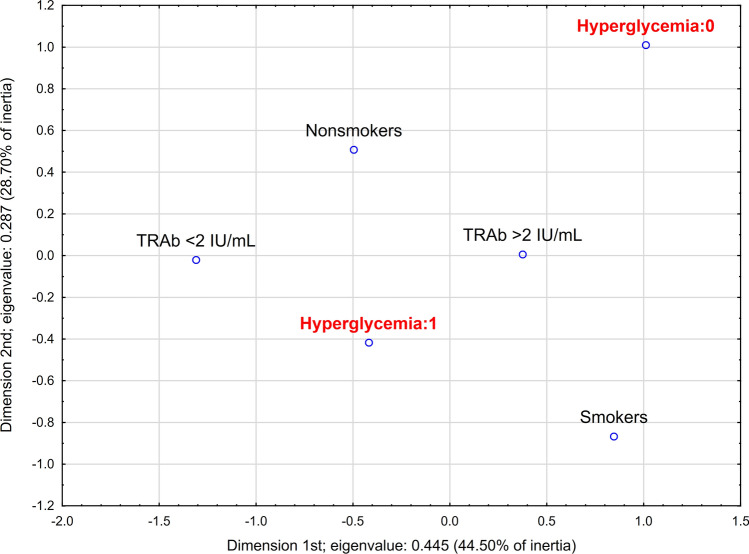
Multidimensional correspondence analysis for steroid-induced hyperglycemia in patients with Graves’ orbitopathy—2-dimensional plot. *TRAb* thyroid-stimulating hormone receptor antibodies

Based on the MPA, conclusions can be drawn about the relationship of major complications with qualitative variables. Decisions on the number of MPA dimensions were made based on the scree plots. The steroid-induced aminotransferase increase was most strongly associated with smokers and patients who have not supplemented LOLA (Figs. 2 and 3). On the other hand, normal TRAb levels and non-smoking predisposed to hyperglycemia (Fig. 4). Detailed point parameters are reported in Table 6.

**Table 6:** Detailed parameters of determined points in multidimensional correspondence analysis for steroid-induced aminotransferase increase (presented in Figs. 2 and 3) and steroid-induced hyperglycemia (presented in Fig. 4) in patients with Graves’ orbitopathy

	*x*	*y*	*z*	quality	relative inertia	*x* inertia	*x* Cos^2^	*y* inertia	*y* Cos^2^	*z* inertia	*z* Cos^2
3-dimensional correspondence analysis for steroid-induced aminotransferase increase											
Nonsmokers	− 0.189	− 0.625	0.302	0.834	0.077	0.014	0.056	0.216	0.63	0.054	0.147
Smokers	0.305	1.008	− 0.487	0.834	0.123	0.022	0.056	0.348	0.63	0.087	0.147
GO duration < 1y	− 0.933	0.197	0.167	0.671	0.117	0.23	0.623	0.015	0.028	0.011	0.02
GO duration > 1y	0.668	− 0.141	− 0.12	0.671	0.083	0.165	0.623	0.01	0.028	0.008	0.02
Previous *iv* GCs:0	− 0.476	0.307	0.044	0.767	0.059	0.101	0.539	0.059	0.224	0.001	0.005
Previous *iv* GCs:1	1.133	− 0.731	− 0.104	0.767	0.141	0.24	0.539	0.141	0.224	0.003	0.005
LOLA:0	0.306	0.358	1.928	0.88	0.163	0.011	0.021	0.021	0.029	0.649	0.83
LOLA:1	− 0.068	− 0.08	− 0.431	0.88	0.037	0.002	0.021	0.005	0.029	0.145	0.83
Aminotransferase increase:0	− 0.346	− 0.27	− 0.124	0.589	0.052	0.056	0.34	0.048	0.206	0.011	0.043
Aminotransferase increase:1	0.981	0.764	0.35	0.589	0.148	0.159	0.34	0.136	0.206	0.031	0.043
2-dimensional correspondence analysis for steroid-induced hyperglycemia											
Nonsmokers	− 0.496	0.507		0.859	0.123	0.116	0.42	0.189	0.44		
Smokers	0.847	− 0.867		0.859	0.21	0.198	0.42	0.322	0.44		
TRAb < 2 IU/mL	− 1.31	− 0.021		0.493	0.259	0.287	0.492	0.0001	0.0001		
TRAb > 2 IU/mL	0.376	0.006		0.493	0.074	0.082	0.492	0.00003	0.0001		
Hyperglycemia:0	1.012	1.01		0.844	0.236	0.224	0.423	0.346	0.421		
Hyperglycemia:1	− 0.418	− 0.417		0.844	0.097	0.093	0.423	0.143	0.421		

*GCs* glucocorticoids, *GO* Graves’ orbitopathy, *iv* intravenous, *LOLA* l-ornithine l-aspartate, *TRAb* thyroid-stimulating hormone receptor antibodies

## Discussion

In our retrospective study, we evaluated the safety profile of a non-standard regimen of 1 g intravenous methylprednisolone (MP) administered for three consecutive days in patients with active, moderate-to-severe or sight-threatening GO. In turn, the recommended treatment for these patients is based on intravenous MP administration on a weekly regimen. However, the cumulative dose of MP should be less than 8 g per cycle, and a single dose of MP should not exceed 0.75 g [13].

The novelty of our study is related to the regimen based on a 3-day intravenous administration of methylprednisolone continued by intramuscular injections, that limits the need for hospitalization and decreases the cumulative dose of steroids. The main reason why administration of methylprednisolone in patients with moderate-to-severe GO over the course of the next 3 days is not recommended is a concern for severe adverse events.

We have demonstrated that 3-day pulses of methylprednisolone do not lead to sudden deaths, liver damage, or thrombosis. Our summary indicated that the applied regimen is relatively safe. A necessary condition is a thorough analysis of risk factors, virological examinations, and metabolic studies. Simultaneously, the regimen limits the need for hospitalization to a few days and allows for the continuation of therapy in outpatient settings. Thyroid orbitopathy is a chronic condition that adversely affects the quality of life, among other things, by worsening socioeconomic conditions [20]. Weekly journeys to a reference center, sometimes located hundreds of km away, are burdensome and exacerbate this situation.

Regardless of the treatment regimen, the clinical use of both short-term and long-term GCs therapy is limited by a wide range of side effects, such as hyperglycemia, insulin resistance, hypertension, arrhythmias, liver dysfunction, psychosis and infections [21, 22]. Also, the frequency and severity of these complications depend on treatment duration, dosage, and route of administration [15]. We reported several mild side effects, such as hyperglycemia, slight aminotransferase increase, headache, and facial erythema. No severe complications, including cardiovascular or hepatic injury, were observed.

GCs therapy is frequently linked with hyperglycemia, glucose intolerance, and diabetes development. Indeed, the effect of GCs on glucose metabolism is a consequence of multiple pathways impairment, such as increased hepatic gluconeogenesis and insulin resistance mainly in the liver and skeletal muscles by interfering with the insulin signaling cascade, as well as through the stimulation of lipolysis and proteolysis [22, 23]. Our study observed that more than half of GO patients presented steroid-induced hyperglycemia. Interestingly, concomitant diabetes did not affect the presence of hyperglycemia. Also, a higher proportion of hyperglycemia appeared in non-smoking patients and patients with normal TRAb levels. Patients with GD have a higher risk of hyperglycemia and diabetes [24]. Also, in the hyperthyroid phase, they have a higher mean amplitude of glycemic excursions [25]. What is more, restoration of thyroid function in those patients leads to deterioration of body composition and visceral fat tissue accumulation [26]. Also, GD and GO are conditions of low-grade inflammation with elevation of circulating adipokines and inflammatory markers [27, 28]. TSH receptors are expressed in the visceral fat tissue in patients with GD and GO and correlate with autoimmunity/inflammation [29]. One may suggest that a higher titer of TRAb may stimulate visceral fat tissue secretory function, leading to adipokines increase and insulin resistance. Those mechanisms could explain the observed association between TRAb titer, CAS and glucose levels.

Cigarette smoking is associated with lower food intake and increased energy expenditure. One of the main causes of increased glucose levels during steroid therapy is an increase in appetite. We could speculate that smokers did not experience that effect that alleviated the influence of steroids on glucose metabolism [30].

Glucose level alterations are observed within hours of GCs exposure and seem dose-dependent [31]. Moreover, Liu et al. found that systematic GCs treatment induces hyperglycemia in 32% and diabetes in 19% of nondiabetic patients [32].

In the literature, no cases of acute liver injury have been reported at cumulative GCs doses up to 8 g [33]. Potential mechanisms of hepatocyte damage include direct dose-dependent toxicity, hypersensitivity reactions and induction of viral or autoimmune hepatitis. In turn, the more commonly observed steroid-induced side effects are asymptomatic elevations of aminotransferases [15, 34–36]. Our study found significantly increased ALT and decreased AST after therapy. Moreover, it should be emphasized that only two patients had clinically significant increases in aminotransferases higher than three times the ULN.

However, LOLA supplementation in most patients may be a co-founding factor for these findings. In this group, lower post-treatment levels of both liver enzymes, as well as lower elevations of ALT and a significant decrease in AST, were observed. Previous studies suggest that LOLA administration enhances hepatocytes’ ability to excrete ammonia and improves the functional integrity of the hepatocyte cell membrane. Thus, it prevents hepatocyte damage and the secretion of aminotransferases into the blood [37–39].

Although severe GO course is more common for males, we observed significantly reduced odds of elevated aminotransferases in this group. In this case, the less toxic effect of steroids may result from more intense inflammation. In addition, males are characterized by higher body mass, which results in a lower dose of steroids per kg.

Moreover, smoking is associated with a more severe GO, especially treatment-resistant [40]. Xing et al. concluded that even former smoking is an independent risk factor for impaired response to intravenous GCs [41]. Our study showed that smokers with disease duration longer than a year more often demonstrated elevated aminotransferases. This may be related to more extended therapy in patients with a poorer response [42, 43].

Interestingly, Aktaran et al. observed a positive correlation between TRAb levels and inflammatory signs in GO patients smoking more than 20 cigarettes per day during the GCs therapy. It is speculated that smoking alters the structure of thyrotropin receptors, making it more immunogenic and consequently leading to the production of TRAb that reacts with retrobulbar tissues [44, 45].

In general, GCs cause blood pressure increases, but only a few of our patients reported significant increases in blood pressure during the therapy [15, 46]. Previously, Miśkiewicz et al. noticed significant elevated maximal systolic blood pressure and mean nocturnal blood pressure during the last bolus of methylprednisolone in GO therapy [47].

Previous studies describe only long-term complications related to the cumulative dose of GCs, but do not consider the administration of high boluses in GO therapy. The most common side effect include osteoporosis, osteonecrosis, diabetes, metabolic syndrome, cardiovascular diseases, infections and cataract. It is worth noting that the risk of complications such as osteoporosis or diabetes is proven to be dose-dependent. Therefore, it is recommended that the doses of GCs should be as low as possible and administered for as short a period as possible [48–50].

Importantly, our Department is a reference centre in GO treatment for two Polish voivodships with more than 4.5 million inhabitants (according to “Area and population in the territorial profile in 2023” by Statistics Poland). Our intravenous 3-day regimen of steroid therapy followed by intramuscular injections, minimizes the frequency of regular hospitalizations with the need to travel to larger medical centres every week. Intramuscular maintenance therapy can occur at the patient’s residence, reducing commuting costs to reference centres. This is important for patients with GO who face physical and mental difficulties daily, which also affects their position in the labor market. Often, they cannot keep their jobs for health reasons, which reduces their social and financial quality of life [20, 51].

Among the limitations of the retrospective study, we are aware of the missing data in medical records. Another limitation was the lack of patient mobility regarding follow-up visits, including ophthalmic examinations, often not carried out in our centre, which is a reference centre for two provinces. Due to the subjective character of some complications, it may be speculated that they had not always been recorded in medical records. The relatively high percentage of patients with the sight-threatening GO may result from the urgent mode of admission in this condition to our Department, and it does not reflect the epidemiological data.

## Conclusion

Treatment with high-dose systemic glucocorticoids applied successfully to manage active, moderate-to-severe or sight-threatening Graves’ orbitopathy, leads to mild side effects, mainly manifested by hyperglycemia and elevated liver enzymes. However, the increased aminotransferase values do not indicate steroid-induced severe liver injury. Thus, the treatment should be carried out in specialized centres, and laboratory parameters need to be monitored during the treatment.

## Data Availability

The datasets generated during and/or analyzed during the current study are available from the corresponding author upon reasonable request.

## Abbreviations

AEs: Adverse events
ALT: Alanine aminotransferase
ALT-1: Alanine aminotransferase before steroid therapy
AST: Aspartate aminotransferase
AST-1: Aspartate aminotransferase before steroid therapy
BMI: Body mass index
CAS: Clinical activity score
CI: Confidence interval
CRP: C-reactive protein
DON: Dysthyroid optic neuropathy
fT3: Free triiodothyronine
fT4: Free thyroxine
GCs: Glucocorticoids
GD: Graves’ disease
GO: Graves’ orbitopathy
*im*: Intramuscular
*iv*: Intravenous
*iv* GCs: Intravenous glucocorticoid pulse therapy
LOLA: L-ornithine l-aspartate
MP: Methylprednisolone
MPA: Multidimensional correspondence analysis
OR: Odds ratio
PPI: Proton pump inhibitor
RAI: Radioactive iodine
SE: Standard error
TAO: Thyroid-associated ophthalmopathy
TED: Thyroid eye disease
TgAb: Thyroglobulin antibodies
TPOAb: Thyroid peroxidase antibodies
TRAb: Thyroid-stimulating hormone receptor antibodies
TRAbs: Thyrotropin-receptor antibodies
TSH: Thyroid-stimulating hormone
ULN: Upper limit of normal
